# Central role of hypoxia-inducible factor-1α in metabolic reprogramming of cancer cells: A review

**DOI:** 10.1097/MD.0000000000040273

**Published:** 2024-11-01

**Authors:** Bing Zhu, Lichao Cheng, Baosu Huang, Runzhi Liu, Bin Ren

**Affiliations:** a Affiliated Hospital of Shandong Second Medical University, School of Clinical Medicine, Weifang, China.

**Keywords:** chemotherapeutic agents, chemotherapeutic drug resistance, HIF-1α, metabolic reprogramming

## Abstract

Metabolic reprogramming is one of the characteristics of tumor cell metabolism. In tumor cells, there are multiple metabolic enzymes and membrane proteins to regulate metabolic reprogramming, and hypoxia inducible factor-1α (HIF-1α) can be regulated in transcription, translation, posttranslational modification and other aspects through multiple pathways, and HIF-1α affects multiple metabolic enzymes and membrane proteins during metabolic reprogramming, thus playing a central role in the metabolic reprogramming process, and thus has some implications for tumor therapy and understanding chemotherapy drug resistance. HIF-1α affects a number of metabolic enzymes and membrane proteins in the metabolic reprogramming process, thus playing a central role in the metabolic reprogramming process, which has certain significance for the treatment of tumors and the understanding of chemotherapeutic drug resistance. In this paper, we review the central role of HIF-1α in metabolic reprogramming, chemotherapeutic agents targeting HIF-1α, and chemotherapeutic drug resistance.

## 1. Introduction

A global cancer statistics report published in 2024 states that there were nearly 20 million new cancer cases worldwide in 2022, with an estimated 9.7 million deaths due to cancer, and that according to population projections, there will be 35 million new cancer cases by 2050, making cancer the most serious public health problem facing humankind.^[1]^ Metabolic reprogramming exists in most solid tumor cells and has become one of the characteristics of cancer cells. A variety of substances have been found to regulate the metabolic reprogramming of tumor cells, among which, hypoxia-inducible factor-1α plays a central role in tumor cell metabolic reprogramming.^[2]^ Understanding hypoxia inducible factor-1α (HIF-1α) is of great significance for understanding tumor pathogenesis, exploring novel antitumor drugs and preventing antitumor drug resistance. In this paper, we review the newly discovered central role of HIF-1α in regulating the metabolic reprogramming of tumor cells in recent years and review the current research status of chemotherapeutic drugs using HIF-1α as an entry point.

## 2. Hypoxia-inducible factor-1α plays an important role in tumor cells

**Figure 1.**
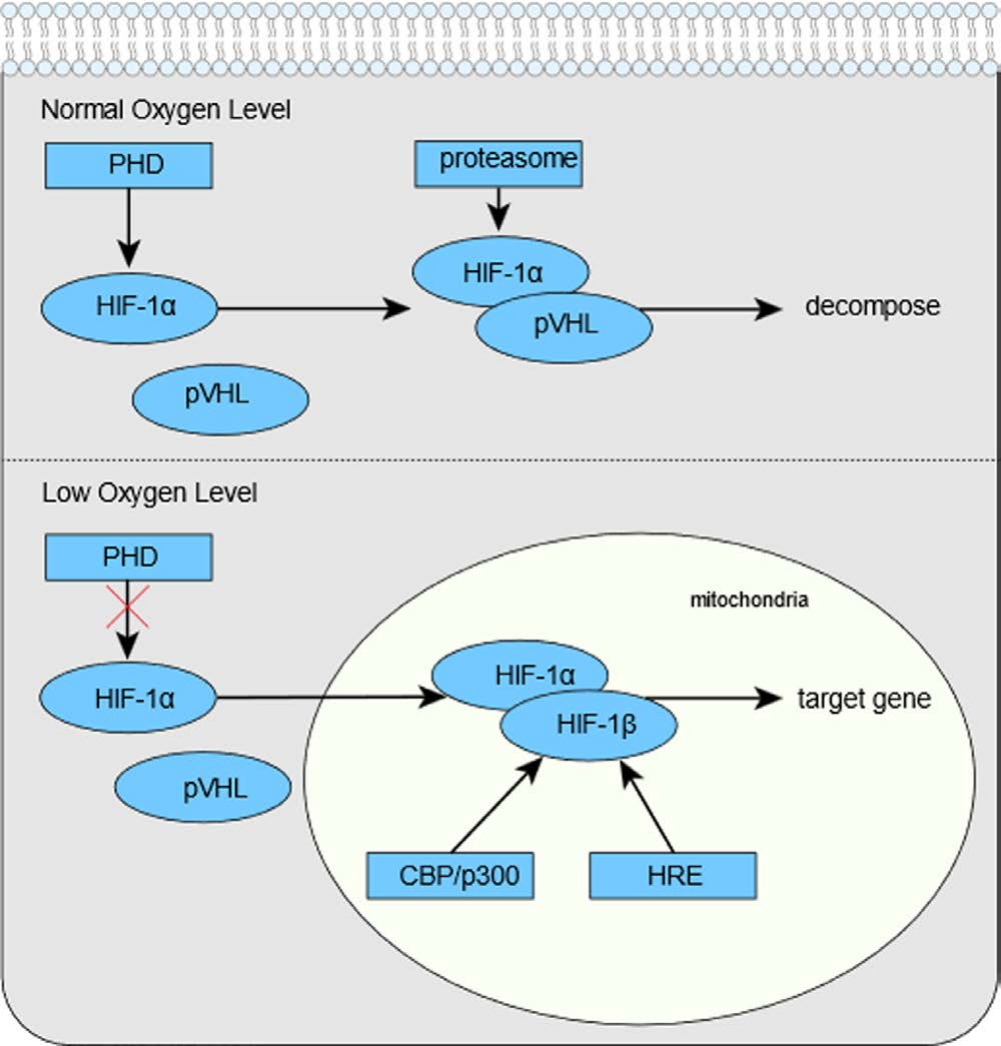
Activation and degradation of HIF-1α. CBP/p300 = the transcriptional activator; HIF-1α = hypoxia inducible factor-1α; HIF-1β = hypoxia inducible factor-1β; HRE = hypoxia response elements; PHD = proline hydroxylase; pVHL = von Hippel-Lindau tumor suppressor protein.

Hypoxia inducible factor-1 is a heterodimeric helix-loop-helix protein consisting of HIF-1α and a constitutively expressed hypoxia inducible factor-1β (HIF-1β). In general, under normal oxygen content conditions, the conserved proline residue of HIF-1α is hydroxylated by proline hydroxylase (PHD), which binds to von Hippel-Lindau tumor suppressor protein (pVHL) and catalyzes its ubiquitination and degraded by the ubiquitination-dependent proteasome. After inhibiting the binding of HIF-1α to pVHL using drugs, a significant upregulation of HIF-1α can be found.^[3]^ However, under hypoxic conditions, PHD is inhibited and HIF-1α cannot be degraded, which causes HIF-1α to aggregate and translocate to the nucleus, where it forms a HIF-1α/HIF-1β heterodimer with HIF-1β. The HIF-1α/HIF-1β heterodimer then binds to the transcriptional activator CBP/p300 and hypoxia response elements to activate transcription of hypoxia-inducible factor-1 target genes (Fig. 1).^[4,5]^ HIF-1α is regulated in a variety of ways, for example, glycerol-3-phosphate dehydrogenase 1 decreases HIF-1α expression by increasing PDH activity, and miR-210-3p regulates PHD activity.^[6]^ The p21 gene can promote HIF-1α transcription at the mRNA level and maintain HIF-1α function, and in turn, HIF-1α can bind directly to the hypoxia-responsive element of the p21 gene promoter to enhance its transcriptional activity.^[7]^ This kind of positive feedback regulation is not uncommon in the regulatory process involved in HIF-1α, and will be mentioned many times in this paper. A very important feature of positive feedback regulation is directionality, and positive feedback regulation in tumor cells will make it continuous in a certain direction, leading to tumor progression. In addition, it has been found that the expression of HIF-1α is also increased in isolated cells under the state of normal oxygen content, which may be related to the increased synthesis of aberrant mRNAs and proteins, which increase the transcriptional activity of HIF-1α.^[8,9]^ For example, p53 proapoptotic protein inhibitors play an important role in regulating HIF-1α signaling under normal oxygen content conditions.^[10]^ pVHL gene mutations bypass the ubiquitination checkpoint and therefore prevent HIF-1α from being degraded by the ubiquitination-dependent proteasome and maintain HIF-1α levels under normal oxygen content conditions^[11]^ HIF-1α is highly expressed and plays an important role in many malignant tumors, including gastric, breast, and bladder cancers.^[6,12,13]^ HIF-1α is highly expressed and plays an important role in many malignant tumors such as gastric, breast, and bladder cancers. It is now believed that HIF-1α plays a key role in the response to hypoxia and is involved in various biological processes such as angiogenesis, epithelial–mesenchymal transition, and metabolic reprogramming.^[14]^ HIF-1α is a key player in the response to hypoxia. At the same time, HIF-1α upregulation also contributes to the maintenance of stem cell-like properties of tumor cells, including the ability of self-renewal and differentiation.^[15]^ In the process of glycolysis, there are multiple biological enzymes, which have crucial regulatory roles in glycolysis, and in the process of aerobic glycolysis in tumor cells, there are multiple pathways to regulate the expression of these biological enzymes to maintain the energy required for the life activities of tumor cells. HIF-1α can promote the expression of catalase genes such as lactate dehydrogenase A (LDHA), hexokinase 2 (HK2) and pyruvate kinase isozyme type M2 (PKM2) in the glycolysis pathway.^[3]^ It can be said that HIF-1α plays an important role in the whole aerobic glycolysis process.

## 3. Metabolic reprogramming of tumor cells and the central role of HIF-1α in metabolic reprogramming

**Figure 2.**
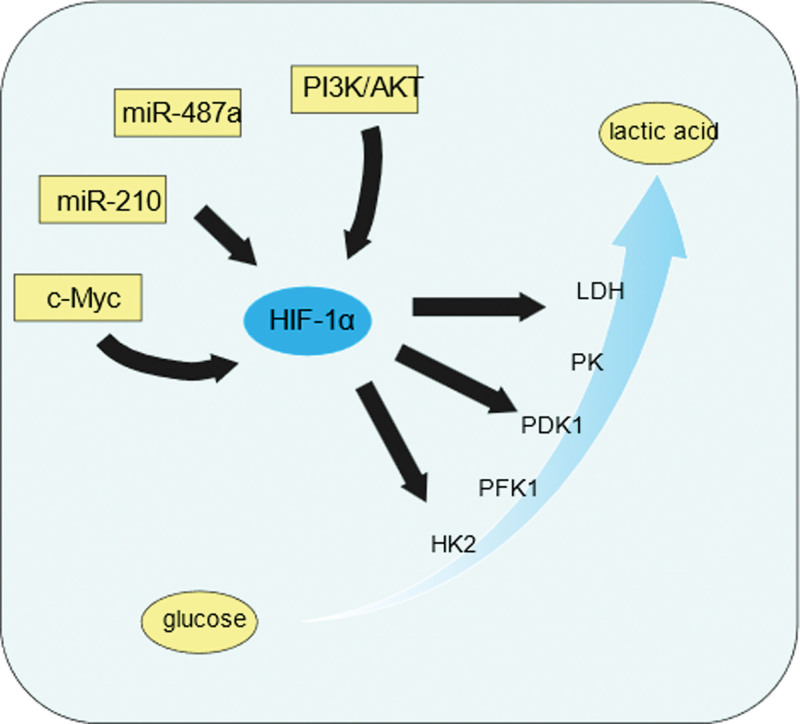
The central role of HIF-1α. HIF-1α = hypoxia inducible factor-1α; HK2 = hexokinase 2; PDK1 = pyruvate dehydrogenase kinase 1; PFK1 = phosphofructokinase 1; PK = pyruvate kinase; LDH = lactate dehydrogenase.

Tumor cells need to consume a large amount of energy to maintain their life activities, adenosine triphosphate (ATP) as a direct source of energy, there are a variety of ways to generate in normal cells, the most important of which is the citric acid cycle and glycolysis, glucose through glycolysis to generate pyruvate, the process of generating a small amount of ATP, under oxygen Under adequate oxygen conditions, pyruvate continues to enter the citric acid cycle and oxidative phosphorylation, generating large amounts of ATP, but under hypoxia, pyruvate is converted directly to lactate. Warburg found that, even in the presence of adequate oxygen, ATP in tumor cells is still generated primarily through glycolysis, a phenomenon known as metabolic reprogramming or aerobic glycolysis. Tumor cells rely on this for much of the energy they need to grow and proliferate. The regulation of this process is extremely complex and diverse, in which HIF-1α participates in and influences the metabolic reprogramming of tumor cells in several ways.^[16]^ For example, circ_03955 can affect the metabolic reprogramming of pancreatic cancer cells through the miR-3662/HIF-1α axis, and in addition, knockdown of miRNA-3662 significantly promotes metabolic reprogramming in hepatocellular carcinoma cells^[17]^ Up-regulation of HIF-1α expression was associated with metabolic reprogramming in triple-negative breast cancer, and metabolic reprogramming was inhibited when HIF-1α expression was down-regulated.^[18,19]^ HIF-1α can induce the expression of multiple glycolytic pathway catalytic enzymes and related substances, and a variety of regulatory factors can regulate the expression of HIF-1α through a variety of pathways and modes, so it can be said that HIF-1α plays a central role in the metabolic reprogramming of tumor cells (Fig. 2), and HIF-1α is like a common “estuary” of multiple rivers. HIF-1α is like a common “estuary” for many rivers, and the study of drugs targeting this key site has a multiplier effect on controlling the metabolic reprogramming of tumor cells.

### 3.1. Hexokinase 2

Hexokinase is the first catalytic enzyme in the glycolytic pathway, which can catalyze glucose phosphate to glucose 6-phosphate, and it is also the first rate-limiting enzyme in the pathway, and it is the “gatekeeper” in the glycolytic pathway, and 4 isozymes have been identified in mammals, and HK2 is one of them. Studies in breast cancer cells have shown that HK2 is regulated by the PI3K/AKT/HIF-1α axis, and when the PI3K-AKT pathway is inhibited, HIF-1α is upregulated, while phosphorylation of AKT leads to upregulation of HIF-1α in tumor cells.^[20]^ circRNF20 is a novel circular RNA that is highly expressed in breast cancer samples, and its overexpression promotes glucose uptake, increases lactate levels, and promotes aerobic glycolysis in breast cancer cells through the miR-487a/HIF-1α/HK2 axis.^[21]^ HIF-1α usually acts as a direct upstream factor of HK2 in the signaling pathway, and can rapidly and directly affect HK2, which guards the entrance of the glycolytic pathway, thus HIF-1α is crucial for the glycolytic pathway. Previous studies have confirmed that HIF-1α is positively correlated with HK2. It has been hypothesized that HIF-1α has a hypoxia-responsive element in the upstream promoter region of HK2. Recent studies have confirmed that HIF-1α can bind directly to HK2 promoter site 1, and have also demonstrated that HIF-1α targets wild-type, not mutant, of site 1.^[21]^ In addition, the promoter regions of both HK2 and phosphofructokinase 1 (PFK1) genes have HIF-1α regulatory elements, and HIF-1α was found to significantly inhibit the down-regulation of HK2 and PFK1 gene expression when up-regulated with drugs.^[16]^ It can be hypothesized that HIF-1α upregulates HK2 by binding to the promoter and activating the transcription of the HK2 gene, which in turn affects the glycolytic pathway.

### 3.2. Glucose transporters

Glucose, as a raw material for glycolysis, needs to be transported into the cell via transmembrane transport in order to undergo glycolysis, a process that relies on the assistance of glucose transporters. Among them, glucose transporter 1 (GLUT1) has a high affinity for glucose and it is significantly up-regulated in malignant tumors such as colorectal cancer, hepatocellular carcinoma, pancreatic cancer, and lung cancer, and there is a significant positive correlation between HIF-1α and GLUT1; in fact, HIF-1α can induce the expression of GLUT1 and affect the tumor cells’ metabolic process of tumor cells.^[22]^ It has been suggested that dysregulation of the PI3K/Akt/mTOR pathway leads to overexpression of GLUT1 and HIF-1α, which in turn affects tumor cell metabolic processes.^[23]^ Genistein can directly down-regulate HIF-1α to inactivate GLUT1, thereby inhibiting glycolysis and inducing mitochondrial apoptosis in hepatocellular carcinoma cells.^[24]^ HIF-1α also up-regulates GLUT1 and LDHA and promotes glycolysis by inducing p21 protein.^[7]^ HIF-1α also upregulates GLUT1 and LDHA and promotes glycolysis through induction of p21 protein. In addition, GLUT1 expression can be regulated through the miR-210-3p/GPD1L axis, and, a positive feedback loop exists between miR-210 and HIF-1α.^[6]^ This indicates that HIF-1α has a certain regulatory effect on the miR-210-3p/GPD1L axis, and due to the existence of positive feedback regulation between HIF-1α and miR-210, this means that the regulation of glycolysis by HIF-1α through this axis can be accomplished more rapidly. It can be seen that HIF-1α can regulate GLUT1 in a variety of ways, and the up-regulation of GLUT1 allows tumor cells to take up enough metabolic raw materials (glucose) to supply the enormous energy needed for their own metabolism. However, a large amount of glucose into the tumor cells will inevitably be limited by the mitochondria as well as the limited amount of oxygen, leading to the limitation of the citric acid cycle and oxidative phosphorylation process, and the excess glucose can only be subjected to glycolysis, which is exactly in line with the metabolic reprogramming of the tumor cells. At the same time, the positive feedback regulation of this process also allows for a more rapid regulation of glycolysis. However, it has also been pointed out that GLUT1 expression is not regulated by HIF-1α, but the expression of GLUT1 in tumor tissues is significantly upregulated compared with normal tissues, which may be related to the postexpression modification of GLUT1.^[25]^ This may be related to the post-glut1 expression modification.

### 3.3. Lactate dehydrogenase and pyruvate kinase

Pyruvate kinase is a key enzyme in the glycolytic pathway that catalyzes the conversion of phosphoenolpyruvate to pyruvate, which is then catalyzed by lactate dehydrogenase to lactate. As mentioned earlier, prolyl hydroxylase indirectly leads to the degradation of HIF-1α, whereas lactate can increase the stability of HIF-1α by inhibiting prolyl hydroxylase.^[26]^ In addition, lactic acidosis stabilizes HIF-1α and induces angiogenesis through overexpression of vascular endothelial growth factor.^[27]^ HIF-1α can upregulate the Na^+^–H^+^ reverse transporter and excrete H^+^ outside the cell, thus making the external environment of tumor cells acidic, which is associated with poor tumor prognosis.^[11]^ Pyruvate produced by glycolysis can induce the expression of HIF-1α, and HIF-1α can promote the expression of glycolytic enzymes such as PKM2, HK2, and LDHA, thus promoting glycolysis in tumors and forming a positive feedback of tumor progression.^[28]^ Kinesin family member 20A upregulates LDHA and PKM2 in colorectal cancer cells through the c-Myc/HIF-1α axis, which in turn enhances metabolic reprogramming in colorectal cancer cells.^[29]^ Long-stranded noncoding RNAs upregulate HIF-1α in glioblastoma to enhance PKM2 and LDHA expression in cancer cells^[30]^ The HIF-1α/BREF/PKM2 axis promotes glucose metabolism in bladder cancer cells.^[31]^ It can be found that multiple regulatory factors regulate metabolic reprogramming through HIF-1α, PKM2, and LDHA always seem to be increased or decreased at the same time, but since LDHA catalyzes a reversible reaction, it plays an important role in both glycolysis and gluconeogenesis, and tumor cells undergo metabolic reprogramming with a large amount of lactic acid accumulation, but after the regulation of HIF-1α, the tumor cells are still heading towards the generation of lactate, which shows the important role of HIF-1α for metabolic reprogramming, in addition, this phenomenon may be related to the aforementioned positive feedback regulation.

### 3.4. Phosphofructokinase 1

During glycolysis, PFK1 can catalyze fructose 6-phosphate to generate fructose 1,6-bisphosphate, which is the second phosphorylation reaction in the glycolytic pathway, as well as the key rate-limiting step in the glycolytic pathway. 6-Phosphofructokinase-2/fructose-2,6-bisphosphatase 3 (6-phosphofructokinase-2/fructose-2,6-bisphosphatase 3, PFKFB3) can phosphorylate fructose 6-phosphate to fructose-2,6-bisphosphate, which in turn activates PFK1 and increases glycolytic flux in cancer cells. Metformin-induced down-regulation of HIF-1a significantly inhibits 6-phosphofructokinase-2, a potent allosteric activator of PFK1, in hepatocellular carcinoma, and studies have confirmed that metformin can inhibit glycolysis via the HIF-1α/PFKFB3/PFK1 pathway.^[32]^ In addition, PFKFB3 significantly upregulated the expression level of HIF-1α in cancer cells.^[33]^ There may be an as yet undefined positive feedback mechanism between PFKFB3 and HIF-1α that can accelerate tumor progression.

### 3.5. Pyruvate dehydrogenase kinase 1 (PDK1)

As mentioned earlier, HIF-1α can increase glycolytic flux in several ways. Under normoxic conditions, pyruvate, a product of glycolysis, is catalyzed by pyruvate dehydrogenase to acetyl coenzyme A and then participates in the citric acid cycle, which is a key step connecting the glycolytic pathway to the citric acid cycle, while PDK1 degrades pyruvate dehydrogenase. HIF-1α can directly activate the expression of the PDK1 gene to restrict pyruvate from entering the citric acid cycle. In addition, HIF-1α can reduce the number of mitochondria in cells.^[34]^ The citric acid cycle needs to be in the mitochondria in order to proceed normally, and a decrease in the number of mitochondria naturally reduces or even stops the citric acid cycle. At the same time, PDK1 can reduce the ubiquitination level of HIF-1α and improve the stability of HIF-1α.^[35]^

## 4. HIF-1α and chemotherapeutic drugs and chemotherapeutic drug resistance

Many studies have shown that understanding the metabolic reprogramming of tumor cells is fundamental to understanding tumor drug resistance and developing chemotherapeutic agents.^[36,37]^ Tumor cell resistance has always been a major issue to be faced during tumor therapy, whether it is the use of classical drugs or the development of new drugs, tumor resistance is a key issue that needs to be solved urgently. It has been pointed out that HIF-1α can also activate a variety of pathways that enhance tumor cell resistance.^[38]^ And HIF-1α is at the core of tumor metabolic reprogramming, which speculates that understanding HIF-1α is the key to understanding tumor drug resistance and developing chemotherapeutic drugs. Indeed, many studies have been conducted to confirm the effectiveness of anti-HIF-1α drugs. FAM83A-AS1, a long-stranded non-coding RNA, can inhibit glycolysis by suppressing HIF-1α, thereby promoting the proliferation and migration of lung adenocarcinoma, a finding that provides a new target for cancer therapy.^[3]^ In triple-negative breast cancer, coenzyme Q_0_ can inhibit aerobic glycolysis by inhibiting HIF-1α, HK2, LDHA, and GULT1, both under normoxic and hypoxic conditions.^[19]^ As previously described, genistein inhibited glycolysis and induced mitochondrial apoptosis in hepatocellular carcinoma cells by down-regulating HIF-1α; moreover, genistein reversed chemoresistance in hepatocellular carcinoma.^[24]^ It is evident that HIF-1α seems to be involved in the development of drug resistance in tumor cells. In a recent in vitro cell xenograft study, the levels of HIF-1α and GLUT1 were found to be negatively correlated with sensitivity to 5-fluorouracil, and high levels of HIF-1α and GLUT1 were more resistant to chemotherapy with 5-fluorouracil compared to low levels of HIF-1α and GLUT1, which is basically in line with the situation of chemotherapeutic drug resistance in the clinic.^[39]^ As mentioned earlier, PKM2, as a downstream factor of HIF-1α, promotes aerobic glycolysis, and it has been pointed out that PKM2 is highly expressed in chemoresistant cells of colorectal cancer and that chemoresistant cells have an extremely strong glycolysis and ATP production capacity.^[31]^ These findings side by side confirm the involvement of HIF-1α in the development of chemotherapeutic drug resistance, and it is worth noting that HIF-1α does not cause drug resistance in tumor cells in only one way. For example, upregulation of p53 pro-apoptotic protein inhibitor can lead to drug resistance in leukemia, breast cancer, hepatocellular carcinoma, ovarian cancer, renal malignancies.^[10]^ Since HIF-1α plays a central role in aerobic glycolysis and is involved in the development of chemotherapeutic drug resistance, then anti-HIF-1α chemotherapeutic drugs may have great therapeutic potential for drug-resistant individuals in chemotherapeutic drug development and clinical trials. Recently, it has been found that inhibition of the HIF-1α/GLUT1 pathway can effectively increase the sensitivity of hepatocellular carcinoma to 5-caffeoylquinic acid.^[40]^ This finding provides a new idea to solve the drug resistance problem faced in chemotherapeutic drug treatment, such as the combination of chemotherapeutic drugs and anti-resistant drugs or alternating application and other programs, and this idea also needs to be confirmed by corresponding studies. Previous studies have confirmed that sorafenib inhibits HIF-1α protein synthesis.^[41]^ However, in the process of clinical treatment, sorafenib will also appear drug resistance, this situation, although there is no accurate data to support, but from clinical experience, the incidence of drug resistance of sorafenib is extremely high. This shows that although HIF-1α is involved in the occurrence of chemotherapeutic drug resistance, it is obviously not enough to use HIF-1α as a target to prevent the occurrence of chemotherapeutic drug resistance, and an in-depth and comprehensive study of the mechanism of resistance occurrence is needed.

In summary, HIF-1α plays a central role in the metabolic reprogramming of tumor cells and the occurrence of chemotherapeutic drug resistance, HIF-1α can regulate a variety of enzymes in the process of metabolic reprogramming through a variety of pathways, and the regulatory process is diverse, but all of them cannot be separated from HIF-1α, the drugs targeting HIF-1α have achieved preliminary results in antitumor and antidrug resistance, but there are still a large number of problems However, there are still a lot of problems that need to be solved, and this also requires more in-depth research, but in terms of chemotherapy and chemoresistance, HIF-1α still has great potential and deserves further research.

## Author contributions

**Conceptualization:** Bing Zhu, Lichao Cheng, Bin Ren.

**Funding acquisition:** Bing Zhu, Lichao Cheng, Bin Ren.

**Investigation:** Bing Zhu, Lichao Cheng, Bin Ren.

**Methodology:** Bing Zhu, Bin Ren.

**Project administration:** Bin Ren.

**Supervision:** Bin Ren.

**Writing – original draft:** Bing Zhu, Lichao Cheng, Baosu Huang, Runzhi Liu.

**Writing – review & editing:** Bing Zhu, Lichao Cheng, Baosu Huang, Runzhi Liu, Bin Ren.
